# Relationship Between Timing of Peak Height Velocity and Pubertal Staging in Boys and Girls

**DOI:** 10.4274/jcrpe.2007

**Published:** 2015-08-31

**Authors:** Andrea Granados, Achamyeleh Gebremariam, Joyce M. Lee

**Affiliations:** 1 University of Michigan Faculty of Medicine, Department of Pediatric Endocrinology, Michigan, United States of America; 2 University of Michigan Faculty of Medicine, Child Health Evaluation and Research (CHEAR) Unit, Division of General Pediatrics, Michigan, United States of America

**Keywords:** peak height velocity, pubertal staging, growth spurt, adolescent growth

## Abstract

Growth and pubertal development are important health markers. We used the data of a longitudinal growth study on a contemporary sample of US youth to examine the relationship between peak height velocity (PHV) and Tanner staging. We observed a substantial variability in the timing of PHV across Tanner stages, which is an important consideration for clinicians when assessing growth.

## INTRODUCTION

Linear growth and pubertal development are critical markers of the overall health status of a child. Linear growth occurs in tandem with pubertal development, with the activation of the hypothalamic pituitary gonadal axis as the proposed driver of the “adolescent growth spurt” (1). A better understanding of how pubertal staging correlates with the adolescent growth spurt can help clinicians distinguish systemic disease or endocrine dysfunction (2) from normal variants of growth, such as constitutional delay of growth and maturation (3).

The literature on the relationship between the adolescent growth spurt or peak height velocity (PHV) and pubertal staging is relatively sparse, beyond the initial studies performed by Tanner in the mid 1900’s (4). We are unaware of studies that have used contemporary clinical longitudinal cohorts to examine this relationship.

Our objective was to evaluate the relationship between timing of PHV with pubertal development across stages of puberty for boys and girls.

## METHODS

The National Institute of Child Health and Human Development (NICHD) Study of Early Child and Youth Development was a longitudinal study of growth and development that followed children from infancy to 15 years of age starting in 1991. The study recruited a total of 1364 children whose birth was normal, uncomplicated and who were born to healthy mothers.

Height and weight were measured by trained research assistants using standardized procedures (5,6). Height and weight measurements were performed up to 11 times throughout the study, at ages 2, 3, 5, 7, 9-15 years. Since not all children were measured at all time points, we performed imputations using Stata ICE statistical software. After imputing data, we excluded 616 subjects with more than 2 missing measurements after 6 years of age for girls and 8 years for boys. Additionally, we excluded 346 participants since negative height data were recorded in the imputed data. We included 402 subjects with available information on weight, height and pubertal staging.

We used in our study Tanner stage (TS) data for breast development in girls and for genitalia in boys. The measurements were obtained by pediatric endocrinologist or nurse practitioners using visual inspection and palpation. Menarche data was obtained by maternal report via questionnaire.

HV was calculated as the increment in height divided by the difference in age between two consecutive measurements at the study visits. PHV was identified as the largest increase in HV after age 6 years for girls and after age 8 years for boys and the age corresponding to that PHV was tagged as age at PHV.

## RESULTS

Overall there were 402 children, of whom 156 were female and 246 were male. The majority (80.6%) of the subjects were classified as white and the remainder (19.4%) as nonwhite. At 9 years of age, 70.1% of the population had a normal weight (defined as a body mass index (BMI) of 5-84%) and 28.6% were overweight or obese (defined as a BMI ≥85%). The mean age at PHV was 12.1 years (1.4 SD) for females and 13.7 years (1.4 SD) for males. PHV for females was 9.8 cm/year and for boys was 11.3 cm/year. Table 1 shows the percentage of children who achieved PHV across all stages of puberty. The majority of girls (69.1%) had achieved PHV by TS 3 and the majority of boys by TS 4 (58.9%). Additionally, we found that 70.6% of girls had attained their PHV by the time of menarche.

## CONCLUSION

Our study is one of the first to examine the relationship between timing of PHV and pubertal stage in a recent population-based cohort of healthy children. We were surprised by the substantial variability in the timing of PHV, which suggests that PHV is part of a dynamic process that can be expected to occur in later stages of puberty. Current knowledge about the relationship between PHV and pubertal development was previously based on data provided by Tanner in 1976 (4). Tanner reported the relationship of PHV with pubertal stage as a correlation and concluded that age at PHV was related to age at TS 2 for breast in girls and TS 3 in boys (4). However this study was based on a UK population of children growing up in the 1950’s with a limited sample size (6). More recently, Kelly et al (7) published reference ranges for annual PHV for “earlier”, “average” and “later” maturers from a large multicenter, multiethnic contemporary US cohort of children, but the relationship between PHV across all pubertal stages was not evaluated.

Our study can provide reassurance to clinicians and families with concerns for their child’s linear growth. For example, a boy who is TS 3 with no evidence of a growth spurt may still have normal growth because the majority of boys do not reach their PHV until TS 4.

There were limitations to our study. First, we had only yearly measurements of height, weight and pubertal status from 9-15 years of age. Second, we did not have information about adult height for all participants since they were only followed up to 15 years of age. Third, TS, although performed by trained nurse practitioners and endocrinologists by palpation and visual inspection, is a somewhat subjective measurement that may lead to TS misclassification. For instance, differentiating between TS 1 and 2 (enlargement of testis and slight thinning and reddening of the scrotal skin) and between TS 4 and 5 (further scrotum enlargement of 12-20 cm vs. >20 cm and darkening) could explain why PHV is observed in our cohort at TS 1 or 5, not typically expected in the clinical setting. Finally, we did not account for factors that may influence linear growth and pubertal timing such as obesity, which can accelerate puberty in girls (8) but may have mixed effects in boys (9).

This study emphasizes the considerable variation in timing of PHV as it relates to pubertal stage. More empiric studies of growth in contemporary cohorts of children are needed to guide pediatricians in the overall assessment of linear growth and pubertal development.

## Figures and Tables

**Table 1:**
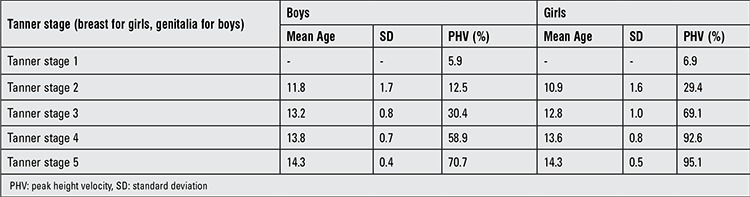
Percentage of children who achieved peak height velocity across all stages of puberty
